# Correlations in Joint Spectral and Polarization Imaging

**DOI:** 10.3390/s21010006

**Published:** 2020-12-22

**Authors:** Guillaume Courtier, Pierre-Jean Lapray, Jean-Baptiste Thomas, Ivar Farup

**Affiliations:** 1 Institut de Recherche en Informatique, Mathématiques, Automatique et Signal, Université de Haute-Alsace, F-68100 Mulhouse, France; guillaume.courtier@uha.fr; 2 Department of Computer Science, Norwegian University of Science and Technology, 2806 Gjøvik, Norway; jean.b.thomas@ntnu.no (J.-B.T.); ivar.farup@ntnu.no (I.F.)

**Keywords:** spectral imaging, polarization imaging, data analysis, correlation analysis

## Abstract

Recent imaging techniques enable the joint capture of spectral and polarization image data. In order to permit the design of computational imaging techniques and future processing of this information, it is interesting to describe the related image statistics. In particular, in this article, we present observations for different correlations between spectropolarimetric channels. The analysis is performed on several publicly available databases that are unified for joint processing. We perform global investigation and analysis on several specific clusters of materials or reflection types. We observe that polarization channels generally have more inter-channel correlation than the spectral channels.

## 1. Introduction

Two branches of unconventional imaging are spectral imaging and polarization imaging. In general, those two approaches are considered independently. When they are considered together, it is often because one modality is a noise for the other, like the polarization effect compensation for spectroscopy [1,2] or wavelength-shift correction for polarization optics [3,4]. Mixing Spectral and Polarimetric Imaging (SPI) is an active emerging research area [5], as it enables a more complete capture of a scene than spectral or polarimetric imaging. Recent work demonstrated the benefits for applications, like computer vision [6,7] or computer graphics [8,9].

Technology advances enable the snapshot acquisition of several spectral and polarization data of the same scene. One technology that has been recently developed is what we call the Generalized Filter Arrays imaging (GFA), which extends the concept of Colour Filter Arrays (CFA) [10] to Spectral Filter Arrays (SFA) [11,12] and Polarization Filter Arrays (PFA) [13]. PFA comes historically in between the CFA and SFA, and aims at filtering the electromagnetic waves relatively to specific polarization directions. The Color Polarization Filter Arrays (CPFA), which has a practical commercial instance in the Sony IMX250 MYR, is one interesting tentative tool for fusing those two branches [14]. A spatial modulation on the focal-plane array permits sampling the intensities of the light field through 12 combined channels; four polarization angles of analyzing uniform-distributed between $0^{\circ }$ and $180^{\circ }$ [15], and three color filters arranged in a Quad Bayer [16] configuration. Because one pixel senses only one channel, computational imaging is used in order to optimize the captured image through an imaging pipeline [17], e.g., demosaicing to reconstruct the spatial resolution. Indeed, one can reconstruct the resolution of the images while using prior knowledge regarding the scene statistics. This is very similar to color and spectral imaging [18,19,20,21,22] and polarization imaging [23,24] based on GFAs.

With the fusion of imaging modalities into one unique imaging setup, it is important to collect prior knowledge regarding image statistics, adapting demosaicing methods to the case of CPFA, and to define an imaging pipeline from sensor design to standardized data representation.

In this article, we investigate the image statistics of joint polarization and spectral information. We implement an experimental protocol and compute the correlation coefficients over several imaging scenarios, involving either diffuse or specular reflection and material clusters. We eventually formulate recommendations in order to help to design performing algorithms for machine vision, help to design sensors of better performance, and help to design adequate imaging pipeline. In Section 2, we defined the sensor and its related data. Subsequently, we describe the experiment used for statistical investigation of data in Section 3. In Section 4, we analyze the results and formulate general observations, before concluding.

## 2. Joint Spectral and Polarization Imaging

### 2.1. Filter Array Imaging

For a general case, light is composed of several wavelengths λ and several polarization states β, so the radiant signal I(λ,β), results from an infinite combination of frequencies and polarization states.

In the sensor case, we characterize the sensing procedure versus the spectral sensitivity by wavelength and versus the Stokes formalism for the polarization state. The Stokes formalism is employed in order to describe the state of polarization in an efficient way [25,26] and it is often visualised within the Poincaré Sphere [27]. In this work, we are only interested by the linear behavior of polarization of the reflected light, thus we do not need to model polarization with the Mueller formalism [28].

The spectral and polarimetric acquisition only detects particular wavelengths λ and particular polarization angle β. This selection is performed according to a detector with given spectral sensitivities and given polarizers, which filter the radiant signal I(λ,β) with filtering functions *f*, and gives output values $\rho_{f}$. In fact, this is often separated in two tracks. One track excludes polarizer and filters the wavelength, and the other track is based on a polarizer and it can exclude the filtering of wavelengths. If there is no polarization filter, then the filtering function *f* is only filtering wavelengths. In that case, all of the polarization states contribute to $\rho_{f}$. If there is no wavelength filter, then *f* is only a linear polarizer, such that all wavelengths contribute to $\rho_{f}$. In the case of one filter that combines a given spectral transmittance and a polarization direction, the detected signal $\rho_{f}$ results from a combination of filters, such as f(λ,β)={c(λ),p(β)}, where *c* indexes the spectral channel and *p* indexes the polarization channel.

For example, the Sony IMX250 MYR is a combination of three spectral filters (c∈R,G,B) and 4 linear polarizers ($p\in {\left\{0,45,90,135\right\}}^{\circ }$). In total, 12 filtering functions *f* exist, which result from the k-combinations of the spectral filters with the polarization filters. At every pixel position, the camera captures the information through only one filtering function, i.e., one pair of spectral and polarization filters. In order to obtain the fully resolved image, i.e., the vector ρ of 12 values per pixel position, we can apply a demosaicing algorithm and estimate I(λ,β) at each spatial position. In the rest of the document, for fluid reading, we will refer to each channel as cp, e.g., R0 for $\left\{R\left(\lambda \right),O^{\circ }\left(\beta \right)\right\}$.

### 2.2. Reflection Model

The dichromatic reflectance model [29] assumes that the reflection of light is composed of a diffuse component (sub-scattering and surface roughness) and a specular component (direct surface reflection). The spectral distribution of the specular reflection component preserves the spectral distribution of the incident light, in the general cases, according to [29]. The diffuse component of the reflection keeps the spectral characteristic of the object multiplied by the light source [29]. The diffuse component is often assumed to be unpolarized. Contrarily, the specular component is partially polarized. This effect is very different within two main classes of materials that exhibit a large difference in their Fresnel reflection coefficients: metal and dielectrics [30], where the typical coefficient plots are shown in Figure 5 in [6].

We however cannot tell a priori how c(λ) and p(β) are mutually dependent. This could be answered based on measurements and on the characterization of the sensing elements and is not the scope of this work. On another hand, natural scenes might exhibit some specific correlations in wavelengths and polarization states depending on the type of material or the type of reflection involved. We can investigate the difference of correlations among the $\rho_{1-12}$ values. To analyze those correlations, we perform statistical analysis on a large body of observations, so it involves different reflection modes and types of materials. This is what we present in the next section.

## 3. Experimental Protocol

**Figure 1:**
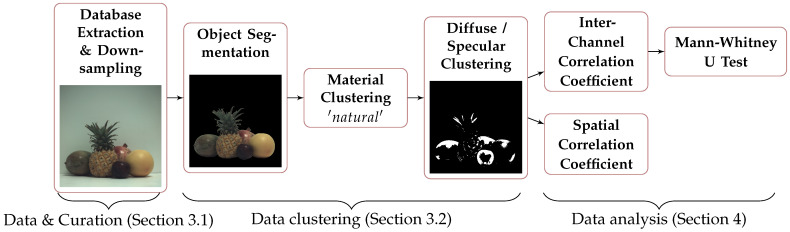
Experimental pipeline. “Data & Curation”, “Data clustering”, and “Data analysis” refer to the Section 3.1, Section 3.2 and Section 4.

We prepare and unify data from different sets to be processed in a batch. Figure 1 shows the different steps. In this Section, we describe the database and the curation of data in Section 3.1, and the classification of data in Section 3.2.

### 3.1. Data & Curation

The first step of our experimental pipeline (Figure 1) addresses the collection and curation of data. To our knowledge, there are currently three databases of joint spectral and polarization images in the literature [31,32,33], but only two are available. Table 1 presents the characteristics of the available databases. The polarization states, with four polarization angles of analysis p∈{0,45,90,135}, are recovered using a division-of-time procedure, i.e., by rotating a uniform polarization filter in front of the camera.

For the spectral filtering, the technology used is the Bayer filter for the two databases. The data from Lapray et al. has six spectral bands, so we converted them into RGB by selecting three bands, followed by a linear colorimetric transform. In general, the spectral characteristics of the RGBs from the two cameras are different, and this may have an impact in our spectral analysis later. However, in this work, we consider that RGB is a standard representation for color image, and we ignore the differences that are related to their spectral characteristics.

The images available in the databases have been demosaiced. In order to mitigate any errors that are introduced by the spatial interpolation, we downsampled all of the images to reduce their size to 0.5 times the size of the original image. Simulating an optical linear filter, a bilinear interpolation, i.e., a weighted average over a 2×2 pixel neighbourhood, is used for the downsampling.

**Figure 2:**
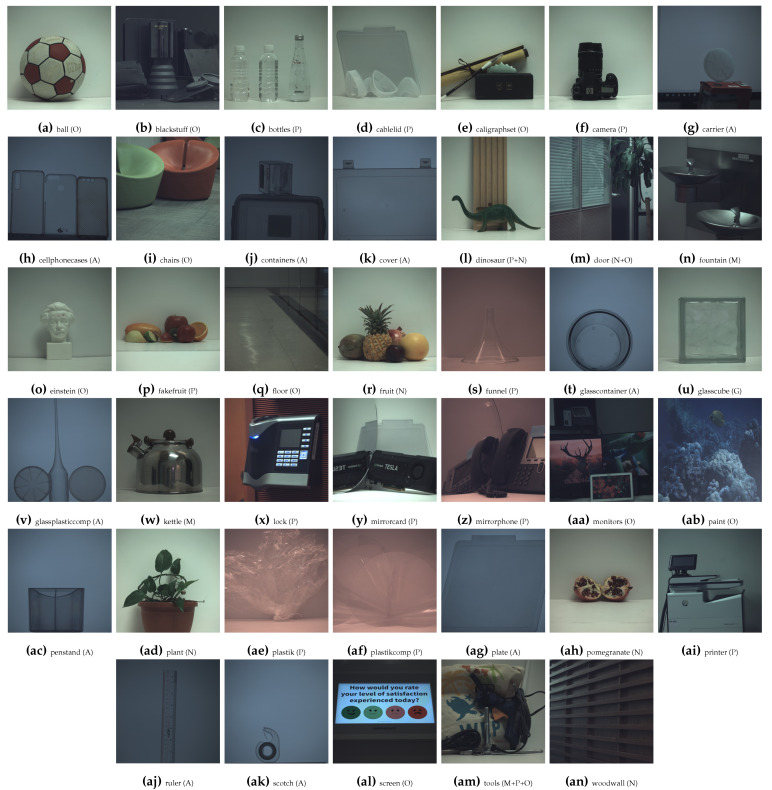
Visualization of the 40 scenes of the data from Qiu et al. [31] by alphabetical order. The material clustering for each scene is represented by the acronym: M (Metallic), N (Natural), A (Active), P (Plastic), G (Glass), and O (Other).

**Figure 3:**
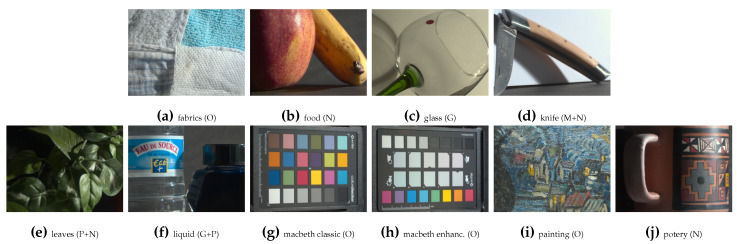
Visualization of the 10 scenes of the data from Lapray et al. [32] by alphabetical order. The material clustering for each scene is represented by the acronym: M (Metallic), N (Natural), A (Active), P (Plastic), G (Glass), and O (Other).

Finally, the data are composed of 12-band images, where each pixel contains a 12-elements vector ($\rho_{1-12}$). The visualization of images is shown in Figure 2 and Figure 3, with their total intensity RGB representation based on each spectral band $S_{0}$ Stokes component [25], such as in Equation 1 of [31]. The scenes consist of different types of material, like manufactured objects that are made of plastic, glass, or metal, or natural objects that are made of organic materials. Illumination is not polarized (i.e., passive polarization imaging), except for 10 scenes with polarized backlight. This last configuration is often employed in order to analyze transparent materials through the photoelasticity experiment [34]. Polarized backlight illumination are used in the scenes of Figure 2g,h,j,k,t,v,ac,ag,aj,ak.

### 3.2. Data Clustering

As previously described in Section 2, we want to study the correlations within several scenarios, based on the material type and the mode of reflection involved (Figure 1). We performed a semantic segmentation for all of the objects in the scenes in order to eliminate the background and only keep observations that belong to the object. The annotation was performed manually, with the help of the polygon lasso tool [35] (Adobe^®^ Photoshop).

After object segmentation, we then visually classified the objects by material clusters, with a relatively well-balanced amount of pixel pertaining to each material. The seven material clusters are defined, as follows: **Total** (all objects—100% of the total amount of object pixels), **Total ∖ {Active}** (82.5%—**Total** without the **Active** cluster), **Metallic** (7%), **Natural** (17.5%), **Active** (i.e transparent object, either glass or plastic, with polarized illumination in background—17.5%), **Plastic** (26.3%), **Glass** (5.4%), and **Other** manufactured objects (26.3%). Some scenes contain pixels that belong to different material clusters (like the dinosaur and piece of wood in Figure 2l), so they have been separated properly. The result of material clustering is shown in Figure 2 and Figure 3. We considered a split in **Active**, **Metallic**, and dielectric from a polarization perspective; we distinguished **Natural** material from manufactured for the spectral properties; and, we added **Plastic** and **Glass** for both the potential transparency or translucency properties and the relevance in bin sorting applications.

In addition to material clustering, we also label each object pixel as being a part of a scene, a part of an object, and also whether it is a specular or a diffuse reflection. Thus, we obtain four different reflection areas: **Scene** (all available pixels, background included), **Object** (background excluded), **Diffuse**, and **Specular**. The classification into specular or diffuse is done using the method described by Nayar et al. [6], where several assumptions are considered: reflections follow a dichromatic model [36]; highlights are specular reflections partially polarized [36]; and, diffuse components are mostly unpolarized. Given the prior assumptions, a threshold per pixel on the Degree of Linear Polarization, DOLP, is applied in order to detect whether the polarization signature of one pixel is sufficient to consider it as specular:

(1) $$\begin{array}{c} \left\{\begin{array}{cc} \mathrm{Spec}=1 & \;\mathrm{for}\;\max_{c\in R,G,B}\left(DOLP_{c}\right)\geq T\;\mathrm{else}\;0,\\ \mathrm{Diff}=1 & \;\mathrm{for}\;\max_{c\in R,G,B}\left(DOLP_{c}\right)<T\;\mathrm{else}\;0, \end{array}\right. \end{array}$$

where *T* is the variable threshold described in [6]. Figure 1 shows an example of the reflection clustering on the fruit scene. The method by Nayar et al. [6] is generally applied only on dielectrics. Nevertheless, in our experience, we also classified **Active** and **Metallic** clusters with this method.

### 3.3. Global Visualization of Data

**Figure 4:**
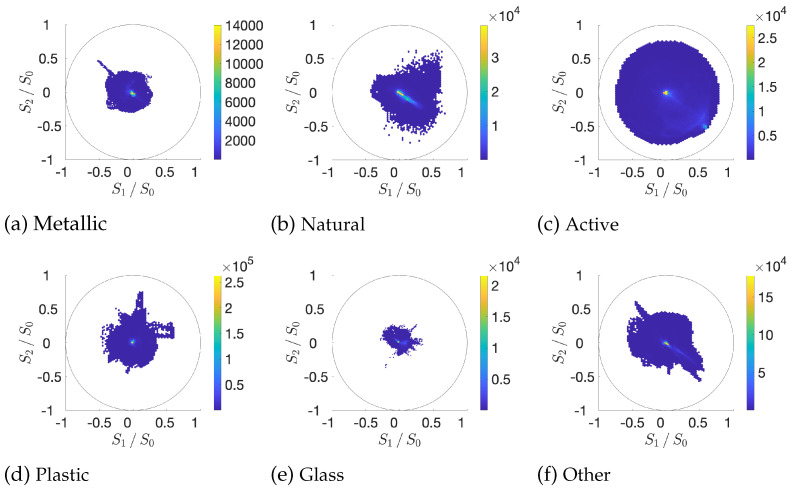
Scatter plots of polarization data for material clusters for the green channel (c=G), approximating the luminance. We also observe similar behaviors on the red and blue channel. Each plot represents the plane of the equator of the Poincaré sphere (latitude is zero for linear polarization). The Stokes component values [25] $S_{1}=\rho_{0,G}-\rho_{90,G}$ and $S_{2}=\rho_{45,G}-\rho_{135,G}$ are normalized with respect to the total irradiance $S_{0}=\rho_{0,G}+\rho_{90,G}$. The center of the circle represents unpolarized data, whereas the unit circle represents fully polarized data. The distance from the center is the degree of linear polarization $DOLP=\frac{\sqrt{S_{1}^{2}+S_{2}^{2}}}{S_{0}}$, and the angle with respect to the origin is 2β, so that the orthogonal polarizations are shown to be diametrically opposed.

Figure 4 shows the diversity of polarization signatures for several materials. The normalized Stokes components for the green channel (c=G) are plotted on the equator plan of the Poincaré Sphere. We see that most of the observations have weak polarization (yellow spots at the center), as can be expected for the majority of man-made and natural materials [37]. The active scenes (with polarized backlight) contain a great variety of angles and degrees of polarization, due to the background polarized illumination. Metallic, Plastic, and Other clusters have very similar shapes. Glass materials exhibit weak polarization when compared to the other clusters. The natural cluster has several sparse observations that have a strong polarization signature; this is due to some noise that is introduced by low irradiance areas (e.g., shadows in Figure 3b,e).

## 4. Data Analysis

### 4.1. Inter-Channel Correlation

In order to analyze the correlation between channels, we computed the Pearson’s correlation coefficient [38,39] (PCC) between all of the 12 available channels $\rho_{u}$ and $\rho_{v}$, $\left(u,v\right)\in {\left\{f_{1},\cdots ,f_{12}\right\}}^{2}$ with Equation (2) [40].

(2) $$PCC\left[\rho_{u},\rho_{v}\right]=\frac{\sum_{i}\left(\left(\rho_{u}^{i}-\mu_{u}\right)\left(\rho_{v}^{i}-\mu_{v}\right)\right)}{\sqrt{\sum_{i}{\left(\rho_{u}^{i}-\mu_{u}\right)}^{2}}\sqrt{\sum_{i}{\left(\rho_{v}^{i}-\mu_{v}\right)}^{2}}}\;,$$

where *i* is the pixel position and $\mu_{u}$ is the mean value of channel $\rho_{u}$.

**Figure 5:**
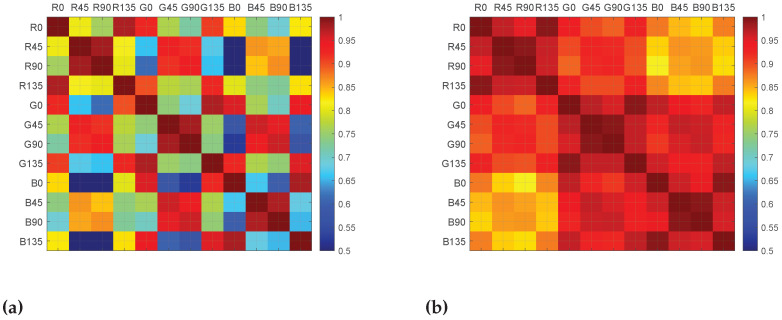
(**a**) **Total Scene** and (**b**) **Total ∖ {Active} Scene** correlation coefficients tables shown in false colors.

The inter-channel correlation coefficients are computed for the four different reflection areas: **Scene**, **Object**, **Diffuse**, and **Specular**. The coefficients are computed for the six material clusters. We obtain 26 correlation coefficient tables, where each table has 12×12 coefficients. Table 2 shows a summary of the correlation results, where the means of coefficients are classified relative to the defined clusters (reflection and material). Cells in orange color have to be taken with care, because the method used for diffuse/specular classification is not valid for **Metallic** and **Active** clusters. By computing the mean, we are smoothing individual differences, but, in Section 4.3, we are looking at the significant differences between distributions. In order to visualize the spectral and polarization correlation independently, each table is rearranged in two ways for convenience: by grouping the polarization bands on the one hand, and by grouping the spectral bands on the other hand. An example is shown in Table 3 and Table 4, with the correlation results for the scenario **Total Object**. We provide the 26 data tables and there visualizations in false colors as supplemental material openly available at [41]. Examples of visualization in false colors are shown in Figure 5.

From the analysis of the coefficients by pair of spectral bands (see **Total Object** results in Table 4), a general behavior is observed: the further apart the spectral bands are, the less the correlation is for the same polarization band. In the same way, by looking at Table 3, the further apart the polarization bands are in term of angle (modulo π), the less the correlation is for a same spectral band. This is expected, since intensity variation follows the Malus law: a modulo π sinusoidal function with respect to the polarization angle. Consequently, the polarization channels are intricately inter-dependent: in the same spectral band, a $0^{\circ }$ pixel value will always be more correlated with a $45^{\circ }$ than with a $90^{\circ }$.

The polarization channels are highly correlated in the **Diffuse** area. This is expected, since the diffuse pixels have been segmented based on the degree of polarization. The spectral correlation is always higher in diffuse reflection than in specular reflection, except for the **Glass** material.

The **Specular** scenario exhibits the lowest correlation values for both polarization and spectral domains. Even in the highlights, which are the areas where polarization is believed to be present, the polarization bands are still highly correlated when compared to the spectral. In fact, in all cases except the **Active** and the **Metallic** materials, the inter-channel correlations are stronger in polarization, in both diffuse or specular zones.

### 4.2. Spatial Correlation

We assess the spatial correlation within a given channel $\rho_{u}$ while using the PCC between the value $\rho_{u}^{i}$ of each pixel *i* and that of its right next-neighbor $\rho_{u}^{i+2}$. We chose i+2 instead of i+1 to mitigate the blur that was introduced by the filter that we applied in Section 3.1. The coefficient is defined, as follows:

(3) $$PCC\left[\rho_{u}\right]=\frac{\sum_{i}\left(\left(\rho_{u}^{i}-\mu_{u}\right)\left(\rho_{u}^{i+2}-\mu_{u}\right)\right)}{\sqrt{\sum_{i}{\left(\rho_{u}^{i}-\mu_{u}\right)}^{2}}\sqrt{\sum_{i}{\left(\rho_{u}^{i+2}-\mu_{u}\right)}^{2}}}.$$

**Figure 6:**
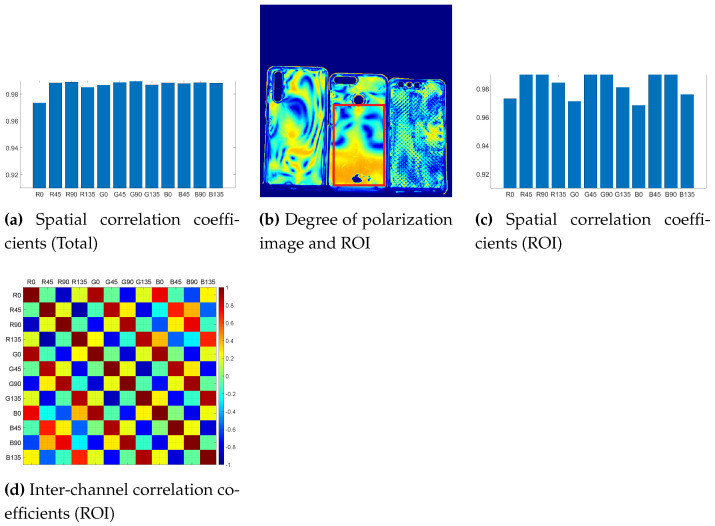
(**a**) Spatial correlation coefficients computed for all of the observations. (**c**) Spatial correlation coefficient computed from an active polarization scene (“cellphonecases” scene in Figure 2h), where the Region Of Interest (ROI) selected is surrounded in red in (**b**). (**d**) Inter-channel correlation computed for the ROI.

Because the amount of edges is low in most natural scenes, and that most of the information is contained in the low frequencies, the spatial correlation among the bands is very high. This is shown in the results presented in Figure 6a. The coefficients have no specific behavior regarding the channel observed. In order to highlight a different behavior, we selected a specific region of interest in one of the **Active** scene, where a large degree of linear polarization is present. Figure 6b shows the area selected, and Figure 6c presents the spatial correlation results on the selected area. In this specific example, we observe that polarization bands correlation are ordered similarly for each of the spectral bands. Some polarization bands have a spatial gradient of intensities significantly different than others, e.g., the $0^{\circ }$ band is the less correlated to the others, whereas the $45^{\circ }$ is the polarization band with the strongest correlation, independent of the spectral band.

### 4.3. Mann–Whitney U (MWU)

We performed a Mann–Whitney U (MWU) test [42] in order to investigate which of the spectral or polarization interchannel correlations is prominent when compared to the other. We did it for all scenarios, using the *ranksum* function in Matlab. The result of the test permits to verify if the medians distributions are within the same range or not. In our case, the null hypothesis (h=0) is when the polarization and spectral correlations are equivalent. The *p*-value is giving us the probability of *h* being true.

Table 3 and Table 4 show the channels that form the two groups of data (one with polarization channels and one with spectral channels) considered in our test, circled by a dark line. We do it for all 26 tables.

Table 5 shows the results for the MWU test. In order to make the results more readable in this table, we define *h*, a binary variable that is equal to one if the two populations of observations are significantly different, zero otherwise. Most of the scenarios have significant differences in their variable distributions (h=1), which invalidates the hypothesis that correlations are uniformly distributed.

From this table, we can revise our observations for Section 4.1. We can then strongly conclude that, if we exclude the **Active** and **Metallic** diffuse/specular scenarios, polarization channels exhibit more correlation than spectral channels. In the case of diffuse reflection, it is always the polarization that is more correlated. This appears to be counter-intuitive when we consider that diffuse reflection tends to depolarize the light. Thus, the polarization angles are randomly oriented, which should have, as result, an extremely low correlation coefficient. However, we are only looking at one particular angle β through an integration process over time, which is compensating for this effect. In the case of metallic objects, the spectral correlation dominates. The cases where the interchannel correlation between polarization or spectral channels is not significantly different (h=0) are on the cluster **Other** and on the specular reflection on **Glass**. For the **Glass**, it is difficult to say that we only have specular component, since an object behind may participate in the radiant information. For the **Other** materials, the difference of characteristics of the objects are so diverse that it is barely useful for performing an analysis. For the **Active** scenes, there is no specific correlation in polarization (Table 2), so the spectral correlation dominates. The **Diffuse** pixels are very little due to the polarized light and the way that we identified the diffused pixels.

### 4.4. Impact on the Development of Spectropolarization Computational Imaging Solutions

This analysis gives us precious indications when it comes to the design or co-design of sensors and pre-processing algorithms, such as demosaicing.

In the co-design of sensors and computational image solution, we want to provide standardized representation of the scene into the image data. Standardized data for color would be encoded in calibrated RGB spaces, spectral data would be encoded as spectral reflectance or relative radiance, and polarization data would be encoded into Stokes vectors. The images should be at a full spatial resolution. In the case of CPFA, one of the limitations is the spatial resolution, and this is addressed by demosaicing. The co-design of the sensor (band distribution) and algorithm will benefit from our analysis. In particular, we have shown that the polarization bands are more correlated than the spectral bands. Thus, the polarization channels should drive the demosaicing process. In other words, better image reconstruction that results from demosaicing can be achieved in the polarization domain, rather than in the spectral domain for dielectric materials. Further investigations must be conducted on metallic surfaces, because our specular/diffuse segmentation of those materials was not very accurate. Similarly, investigations that are related to active light scenes need to be pushed further, because, in this last case, it might be more interesting to demosaic from the spectral information.

## 5. Conclusions

In this article, we investigated and analyzed the statistics of joint spectral and polarization images. We show that the inter-channel polarization information is generally more correlated than for the spectral channels for dielectric materials. Further investigations are required for the case of metallic objects. The case of active lighting is a different specific scenario; it would be interesting to investigate how emerging illumination technologies behave as active lighting. This provides basis for the future development of CPFA imaging solutions.

## Figures and Tables

**Table 1:** Summary of the databases that were used in this work.

Database	Num. Scenes	Full Resolution	Pre-Processing	Spectral Sensing	Polarization Sensing
**Qiu et al. [31]**	40	1024×1024	Averaging 100 images, 2×2 pixel binning	c∈{r,g,b}—Bayer RGB sensor (CMOSIS CMV4000-3E5)	p∈{0,45,90,135}—Rotated linear polarizer (Thorlabs WP25M-VIS)
**Lapray et al. [32]**	10	994×738	Linearization, FPN, PRNU	6-band—Bandpass filters and Bayer RGB sensor (JAI AD-080GE camera)	p∈{0,45,90,135}—Rotated linear polarizer (Newport 10LP-VIS-B)

**Table 2:** Means of correlation coefficients for each pair of reflection/material clusters, either in the Spectral (S) or Polarization (P) channels. The coefficients are calculated from the groups shown in Table 3 and Table 4. Blue cells indicate the cases where spectral correlation coefficient means are higher than polarization. Cells in orange color have to be taken with care.

	Reflection	Scene S	Scene P	Object S	Object P	Diffuse S	Diffuse P	Specular S	Specular P
Material									
**Total**		0.91	0.81	0.86	0.89	0.92	1.00	0.80	0.81
**Total ∖ {Active}**		0.92	0.97	0.86	0.95	0.92	1.00	0.80	0.91
**Metallic**		-	-	0.99	0.96	0.98	0.99	0.98	0.92
**Natural**		-	-	0.86	0.96	0.91	1.00	0.84	0.97
**Active**		-	-	0.90	0.22	0.97	0.99	0.88	0.05
**Plastic**		-	-	0.89	0.98	0.88	1.00	0.88	0.93
**Glass**		-	-	0.97	0.98	0.89	0.98	0.98	0.98
**Others**		-	-	0.85	0.92	0.95	1.00	0.75	0.83

**Table 3:**
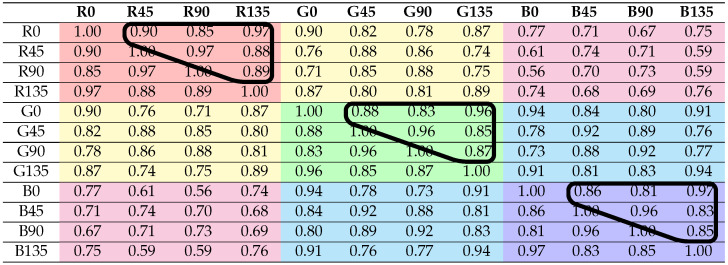
Correlation results for the **Total Object** scenario, over all of the 12 channels grouped by spectral channel. The polarization groups of correlation coefficients (surrounded) are passed to the MWU computation. Channel groups are distinguished by different colors.

**Table 4:**
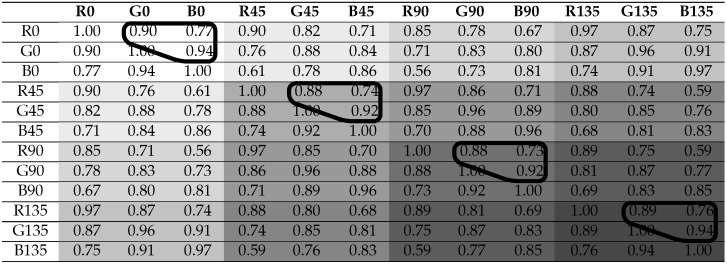
Correlation results for **Total Object** scenario, over all of the 12 channels grouped by polarization channel. Polarization channels are grouped with different grayscale values. The spectral groups of correlation coefficients (surrounded) are passed to the Mann–Whitney U (MWU) computation. Channel groups are distinguished by different colors.

**Table 5:** Mann–Whitney U tests *p*-values and h for the 26 scenarios. h=1 means the rejection of the null hypothesis, whereas h=0 means a failure to reject the null hypothesis at 5% significance level. *P* (Polarization) or *S* (Spectral) indicates which modality has the maximum mean correlation coefficient (from Table 2). Cells in orange color have to be taken with care.

	Reflection	Scene			Object			Diffuse			Specular		
Material		*p*-Value	*h*	Max	*p*-Value	*h*	Max	*p*-Value	*h*	Max	*p*-Value	*h*	Max
**Total**		0.133	0	S	0.385	0	P	0.000	1	P	0.751	0	P
**Total ∖ {Active}**		0.003	1	P	0.001	1	P	0.000	1	P	0.007	1	P
**Metallic**		-	-	-	0.000	1	S	0.000	1	P	0.000	1	S
**Natural**		-	-	-	0.000	1	P	0.000	1	P	0.000	1	P
**Active**		-	-	-	0.000	1	S	0.029	1	P	0.000	1	S
**Plastic**		-	-	-	0.000	1	P	0.000	1	P	0.001	1	P
**Glass**		-	-	-	0.006	1	P	0.000	1	P	0.341	0	P
**Other**		-	-	-	0.122	0	P	0.000	1	P	0.341	0	P
